# [Retracted] Long noncoding RNA UCID sponges miR‑152‑3p to promote colorectal cancer cell migration and invasion via the Wnt/β‑catenin signaling pathway

**DOI:** 10.3892/or.2023.8529

**Published:** 2023-03-20

**Authors:** Li-Bin Sun, Shu-Fen Zhao, Jing-Juan Zhu, Yue Han, Ti-Dong Shan

Oncol Rep 44: 1194–1295, 2020; DOI: 10.3892/or.2020.7670

Following the publication of the above paper, it was drawn to the Editor's attention by a concerned reader that there appeared to be matching data panels comparing between the Transwell invasion and migration assays shown in Figs. 2C and 5C; moreover, one of the data panels shown in Fig. 2D had previously appeared in a paper written largely by different authors (the author ‘T-D Shan’ was held in common) at different research institutes in the journal *Oncotarget* in 2016 [Shan T-D, Xu, J-H, Yu T, Li J-Y, Zhao L-N, Ouyang H, Luo S, Lu X-J, Huang C-Z, Lan Q-S *et al*: Knockdown of linc-POU3F3 suppresses the proliferation, apoptosis, and migration resistance of colorectal cancer. Oncotarget 7: 961–975, 2016]. Finally, an independent investigation of these data in the Editorial Office revealed that, in addition to the data shared between Figs. 2 and 5, there were overlapping data panels both within Fig. 5C and within the wound healing assay data shown in Fig. 3B.

Owing to the fact that the contentious data in the above article had already been published prior to its submission to *Oncology Reports*, and given the number of cases of overlapping data panels both within and between figures in the artce itself, the Editor has decided that this paper should be retracted from the Journal. After having been in contact with the authors, they did not agree with the decision to retract the paper. The Editor apologizes to the readership for any inconvenience caused.

